# Genetic Polymorphisms and Forensic Efficiencies of a Set of Novel Autosomal InDel Markers in a Chinese Mongolian Group

**DOI:** 10.1155/2020/3925189

**Published:** 2020-01-07

**Authors:** Wenqing Zhang, Xiaoye Jin, Yijie Wang, Tingting Kong, Wei Cui, Chong Chen, Yuxin Guo, Bofeng Zhu

**Affiliations:** ^1^Key Laboratory of Shaanxi Province for Craniofacial Precision Medicine Research, College of Stomatology, Xi'an Jiaotong University, Xi'an, China; ^2^Clinical Research Center of Shaanxi Province for Dental and Maxillofacial Diseases, College of Stomatology, Xi'an Jiaotong University, Xi'an, China; ^3^College of Forensic Medicine, Xi'an Jiaotong University Health Science Center, Xi'an, China; ^4^Department of Forensic Genetics, School of Forensic Medicine, Southern Medical University, Guangzhou, China

## Abstract

Insertion/deletion (InDel) markers have been treated as a prospective and helpful aid in the fields of forensic human identifications and biogeography origin researches for the past few years. In this study, we analyzed genetic polymorphisms and forensic efficiencies of 35 InDels in a novel multiplex PCR-InDel panel in a Chinese Mongolian group. All these 35 InDel loci were observed to conform to Hardy–Weinberg equilibrium and linkage equilibrium. The mean values of expected heterozygosity and observed heterozygosity were 0.4788 and 0.4852, respectively. Besides, the interpopulation differentiations and genetic distributions based on 35 InDels found that the Chinese Mongolian group might have closer genetic relationships and similar population genetic structures with East Asian populations.

## 1. Introduction

InDels are length polymorphisms resulting from the insertion or deletion of one or more nucleotides in the genome [1]. In 2002, Weber et al. firstly identified and characterized 2000 human biallelic InDels, which differed greatly in the lengths of the observed alleles, and they also emphasized the usefulness of InDels in genetic researches because of their richness and ease of analysis [2]. Since then, in more and more published studies, InDels have been used for a variety of purposes [3, 4]. InDels have many strengths in forensic analyses: firstly, they are widely distributed across the human genome and commonly display small amplicons which are conducive to the analyses of degraded or dated samples; secondly, the mutation rates of InDels are lower compared with short tandem repeat (STR) loci; thirdly, they have no microvariant products, which could make them more applicable for the interpretation of the mixture. Additionally, they could also serve as ancestry-informative markers (AIMs) for characterizing population substructure and performing biogeographical origin analyses [5–7]. In recent years, more and more studies have found that InDels could be useful in human identification [1], mixed stain identification [8], and so on.

Mongolian, in terms of population size, is the tenth largest ethnic group in China, distributed in Gansu, Qinghai Provinces, Xinjiang Uygur, and Inner Mongolia Autonomous Regions. Some Mongolians also dwell in Liaoning, Jilin, Heilongjiang, and other provinces. The language of Mongolian group belongs to the Altaic family. The main religion of the Mongolian people is Buddhism (http://www.paulnoll.com/China/Minorities/min-Mongolian.html). Nowadays, genetic analyses of the Chinese Mongolian group mainly focused on STR loci, such as 19 X-STR loci [9] and 19 autosomal STRs [10], 22 autosomal STR loci [11], 12 X-STR [12], and 27 Y-STR [13]; besides, Jin et al. used 48 single nucleotide polymorphism (SNP) loci to study genetic relationships among continental populations and Chinese populations including Mongolian group [14]. However, to date, few studies on autosomal InDels in the Chinese Mongolian ethnic group have been conducted.

Previously, we developed a novel multiplex PCR-InDel panel for forensic individual identifications in the Chinese Kazak group and reference populations from East Asia [15]. Here, genetic distributions and forensic efficiencies of these InDels in the Chinese Mongolian group were further investigated. Besides, heat maps of fixation index (*Fst*) and *Nei's genetic distances (D*_*A*_ distances), principal component analysis (PCA), phylogenetic reconstruction, population clustering analysis of the studied Mongolian ethnic group and other reference populations were also constructed to explore their genetic relationships.

## 2. Materials and Methods

### 2.1. Subjects and Sample Collection

We collected a total of 110 bloodstain samples from unrelated healthy Mongolian individuals in China. All subjects signed the written informed consent prior to sampling. This study obtained the approval of the Ethics Committees of Xi'an Jiaotong University Health Science Center and Southern Medical University, China.

### 2.2. PCR Amplification and InDel Genotyping

In this study, the PCR amplification of 35 InDel loci was conducted on a GeneAmp PCR system 9700 thermal cycler (Applied Biosystems, Foster City, CA, USA), following previous descriptions [15]. Then, the PCR amplification products were separated and detected by capillary electrophoresis on the ABI 3500 Genetic Analyzer (Applied Biosystems, Foster City, CA, USA). The allele typing was performed by GeneMapper v3.2 software (Applied Biosystems, Foster City, CA, USA).

### 2.3. Reference Populations

The reference populations of this study were the intercontinental populations [16] and Kazak group [15] reported before, which were African (7 populations), American (4 populations), East Asian (5 populations), European (5 populations), South Asian (5 populations), and Chinese Kazak group. Detailed information of these continental populations are as follows: African populations include ACB, ASW, ESN, GWD, LWK, MSL, and YRI; American populations include CLM, MXL, PEL, and PUR; East Asian populations include CDX, CHB, CHS, JPT, and KHV; European populations include CEU, FIN, GBR, IBS, and TSI; South Asian populations include GIH, ITU, PJL, STU, and BEB. Detailed information of these reference populations was presented in Table 1. And the geographic localization of the Chinese Mongolian and other reference populations was shown in [Supplementary-material supplementary-material-1].

### 2.4. Statistical Analysis

The allelic frequency distributions and forensic statistical parameters which included the values of observed heterozygosity (Ho), expected heterozygosity (He), polymorphism information content (PIC), discrimination power (DP), probability of exclusion (PE), match probability (MP), typical paternity index (TPI), and *P* values for Hardy–Weinberg equilibrium tests (*P* values) and linkage disequilibrium (LD) analyses of 35 InDel loci in Mongolian group were calculated by STRAF online program (version 1.0.5) [17]. A map showing population distributions was plotted by *R* software (version 3.4.5) (https://www.r-project.org/). *D*_*A*_ distances and *Fst* values among the studied Mongolian group and other reference populations were calculated by DISPAN program [18] and Genepop software (version 4.0) [19], respectively. Then heat maps of *D*_*A*_ and *Fst* values of these populations were conducted on pheatmap package (version 1.0.12) by *R* software (version 3.4.5). The PCA of the studied Mongolian group and 27 compared populations was generated by MVSP software (version 3.1). Moreover, PCA of these populations at individual level was conducted by PLINK software (version 1.9) [20], which was visualized by ggplot 2 package (version 3.2.0) of *R* software (version 3.4.5). Beyond that, a multidimensional scaling (MDS) plot [21] was conducted by SPSS software (version 23.0). Additionally, a phylogenetic tree based on *D*_*A*_ distances was established by MEGA software (version 6.06) [22]. The population genetic structure analyses were evaluated by using the STRUCTURE software (version 2.3.4.) [23] and CLUMPP software (version 1.1.2). The appropriate *K* value was assessed by the Structure Harvester online tool (version 0.6.94) (http://taylor0.biology.ucla.edu/structureHarvester/).

## 3. Results

### 3.1. Hardy–Weinberg Equilibrium Tests and Forensic Statistical Parameters of 35 InDels

The *P* values for Hardy–Weinberg equilibrium tests and forensic statistical parameters of 35 InDels were shown in Table 2. From Table 2, the significant differences from *P* values at the 35 InDels were not found after a Bonferroni correction (*P* = 0.05/35 = 0.0014). The PIC values ranged from 0.1190 (rs3054057) to 0.3750 (rs4024564, rs1160964, rs61681053, and rs10556291), with the mean value of 0.3609. The He and Ho values ranged from 0.1276 (rs3054057) to 0.5023 (rs10556291), and from 0.1182 (rs3054057) to 0.6273 (rs3028455), with the mean values of 0.4788 and 0.4852, respectively. The values of MP, DP, PE, and TPI varied from 0.3522 (rs4024564) to 0.7757 (rs3054057), 0.2243 (rs3054057) to 0.6478 (rs4024564), 0.0114 (rs3054057) to 0.3249 (rs3028455), and 0.5670 (rs3054057) to 1.3415 (rs3028455), respectively. Additionally, the frequencies of insertion alleles (+) and deletion alleles (−) ranged from 0.3682 (rs371194629) to 0.9318 (rs3054057), and from 0.0682 (rs3054057) to 0.6318 (rs371194629), respectively. The forensic parameters of cumulative PE and combined DP values of 35 InDel loci were 0.99925 and 0.9999999999999904, respectively.

### 3.2. Linkage Disequilibrium Analyses of 35 InDels

LD tests of these 35 InDel loci in the Chinese Mongolian group were calculated by STRAF online program (version 1.0.5). As shown in [Supplementary-material supplementary-material-1], pairwise InDels were observed to conform to linkage equilibrium after applying a Bonferroni correction (*P* = 0.05/595 = 0.000084), indicating that these 35 InDel loci were mutually independent in the studied Mongolian ethnic group.

### 3.3. Interpopulation Differentiations Based on 35 InDels

**Figure 1:**
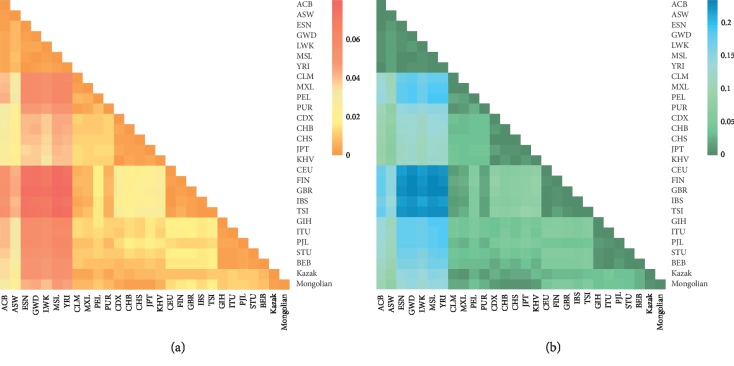
The heat maps of pairwise *D*_*A*_ (a) and pairwise *Fst* (b) values of the Chinese Mongolian group and the 27 reference populations based on the 35 same InDel loci using the *R* software.

Absolute values of insertion allelic frequency differences (*δ*) between the studied Mongolian group and other reference populations were given in [Supplementary-material supplementary-material-1]. Results showed that the studied Mongolian group had relatively low *δ* values (<0.1) with East Asian populations and Kazak group at most loci in comparisons with other reference populations. And then, genetic distances (*D*_*A*_) of the studied group and other reference populations were calculated using allelic frequencies of 35 InDel loci, as shown in Figure 1(a) and [Supplementary-material supplementary-material-1]. As one of the most generally used genetic distances, *D*_*A*_ distance has been used to measure genetic differences of different populations. It is developed on the assumption that genetic drift and mutation events finally lead to genetic differences [24]. As shown in Figure 1(a), the population names at the bottom axis and right vertical axis of the triangle corresponded to the paired populations of each block, which represented the *D*_*A*_ values of the paired populations. The different color scaling on the upper right corner of the plot showed the *D*_*A*_ values ranged from 0 to 0.06 which varied from orange to red. Different colors stood for different levels of *D*_*A*_ values: orange for low *D*_*A*_ values and red for high *D*_*A*_ values. Orange color blocks between the Chinese Mongolian and the Kazak groups as well as five East Asian populations (CDX, CHB, CHS, JPT, and KHV) were observed, indicating that they had close genetic relationships. However, the colors of the blocks between the Mongolian group and some African populations were nearly red, meaning that there were relatively large *D*_*A*_ values between the Chinese Mongolian and these African populations. In addition, we also generated a heat map based on the *Fst* values of the pairwise populations to further measure population differentiations, as shown in Figure 1(b) and [Supplementary-material supplementary-material-1]. Likewise, the population genetic relationships were reflected by the depth of each block's color, which changed from deep green to blue. The closer the color was to deep green, the lower the *Fst* value was, indicating that the genetic differences of pairwise populations were the smaller. We also found that the blocks of the Chinese Mongolian group and five East Asian and Kazak groups showed deep green colors while the Chinese Mongolian group and other intercontinental populations showed light green or blue colors, showing that genetic differentiations between Chinese Mongolian and the five East Asian populations as well as Kazak group were smaller compared to the other reference intercontinental populations.

### 3.4. Principal Component Analysis and Multidimensional Scaling

**Figure 2:**
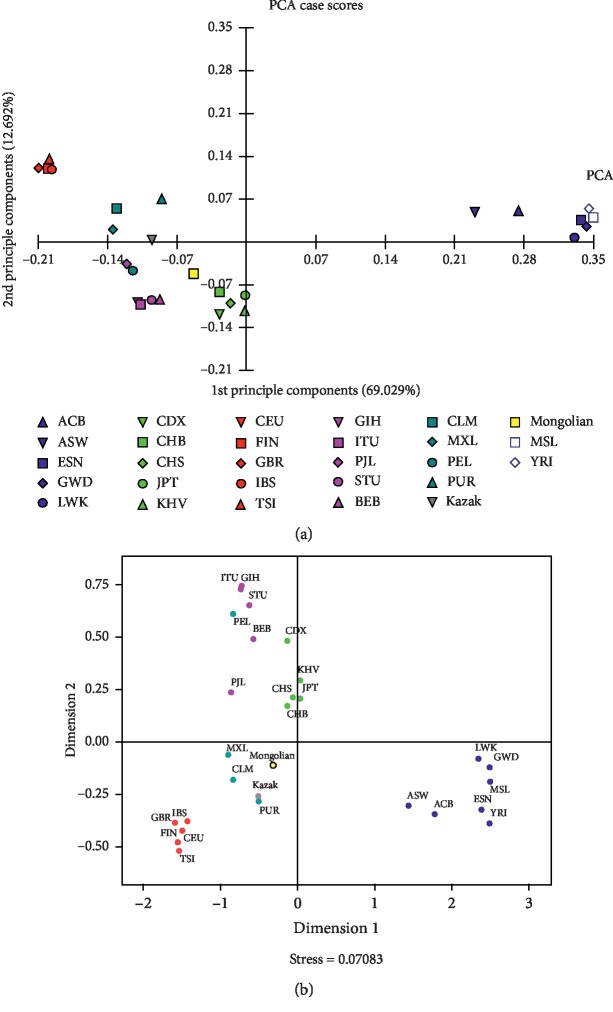
The principal component analysis (a) and multidimensional scaling based on pairwise *Fst* values (b) of the studied Mongolian group and the 27 reference populations. Different symbols represent different populations and different colors represent different geographical regions: blue for African, deep green for American, light green for East Asian, red for European, purple for South Asian, grey for Kazak, and yellow for the studied Mongolian.

The genetic relationships between the Chinese Mongolian and the other compared populations were explored using PCA by the MVSP software (version 3.1). The advantage of the PCA is that it allows graphical representation of multidimensional data with reduced number of dimensions [25]. As shown in Figure 2(a), different continental populations formed the corresponding population clusters which were in line with their geographical origins; however, four admixture American populations were distributed among the European, South Asian, and East Asian populations. Furthermore, we also found that the Chinese Mongolian group was adjacent to five East Asian populations, indicating that the Chinese Mongolian group had closer genetic relationships with these East Asian populations than the other reference intercontinental populations. Moreover, PCA of the studied group and other reference populations at individual level was performed using PLINK software (version 1.90). As shown in [Supplementary-material supplementary-material-1], one point represented a sample, and seven different colors represented five different intercontinental populations and Kazak group as well as the studied Mongolian group. Obtained results revealed that the distributions of some Mongolian individuals were overlapped with the East Asian, Kazak, South Asian, American, and European populations, while African populations separated from them into an independent cluster. The PCA result of individual level was due to the smaller differences in allele frequencies of these 35 InDel loci between the Mongolian group and these four reference continental populations, whereas the larger differences in allele frequencies of these loci between the Mongolian group and African populations in this study.

For further validation, a MDS plot based on pairwise *Fst* values of these populations was generated as shown in Figure 2(b). Similar population distribution patterns were observed in MDS, implying that the Chinese Mongolian and East Asian as well as Chinese Kazak populations had relatively close genetic ties.

### 3.5. Phylogenetic Analysis among the Chinese Mongolian Ethnic Group and 27 Reference Populations

**Figure 3:**
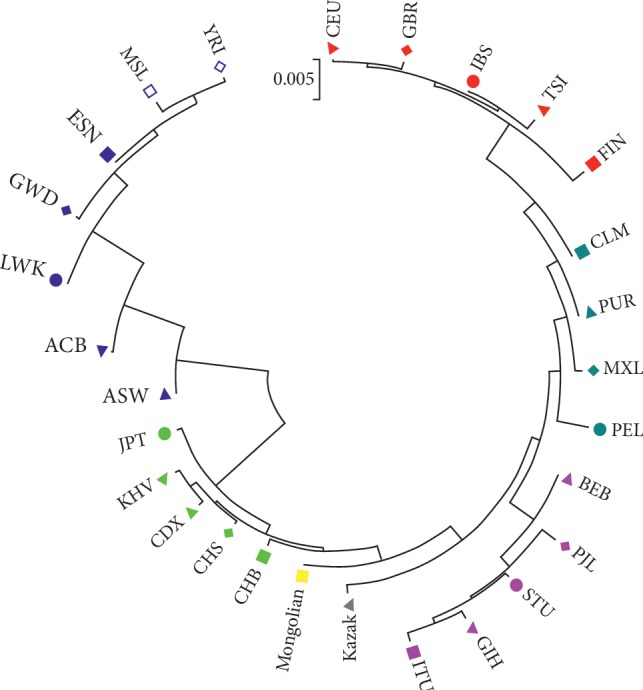
The phylogenetic tree of the Mongolian group and the 27 reference populations by MEGA software (v6.06) based on 35 InDels.

The purpose of phylogenetic analysis is to intuitively infer or evaluate the relationships among different populations [26]. Populations with lower genetic distances commonly form a branch on the phylogenetic tree. We constructed a phylogenetic tree of the Chinese Mongolian and other reference populations by MEGA software (version 6.06). As shown in Figure 3, two main branches could be observed: seven African populations formed a branch; East Asian, South Asian, American, European, Chinese Kazak, and the studied Chinese Mongolian groups formed another branch. At the second branch, five European populations clustered together; four American populations gathered as a subbranch; five South Asian populations gathered as another subbranch; the studied Chinese Mongolian group firstly formed the subbranch with five East Asian populations, and then followed by the Chinese Kazak group, revealing that the Chinese Mongolian group had smaller genetic differentiations with these East Asian and Kazak populations.

### 3.6. Population Genetic Structure Analysis among the Studied Mongolian Group and 27 Reference Populations

**Figure 4:**
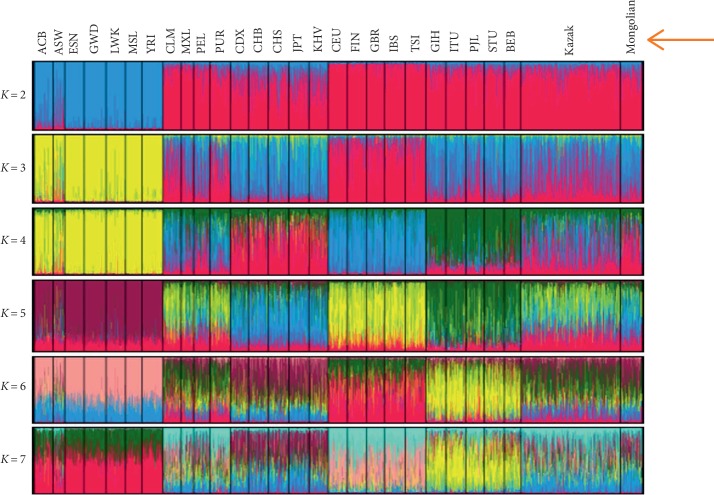
The structure analysis among the studied Mongolian group and the 27 reference populations based on STRUCTURE software (version 2.3.4) and CLUMPP software (version 1.1.2). *K* is from 2 to 7.

In this study, a population clustering analysis method was used to reflect ancestral proportion memberships of the Chinese Mongolian and 27 compared populations with the number of hypothetical populations (*K*) which were assumed from 2 to 7 by using the STRUCTURE software (version 2.3.4.) and CLUMPP software (version 1.1.2). Then the appropriate *K* value was estimated by Structure Harvester (http://taylor0.biology.ucla.edu/structureHarvester/), as shown in [Supplementary-material supplementary-material-1]. Results showed that the appropriate *K* value was 3 for the population data set used in this study according to the appropriate *K* value standard in a previous report [27]. Clustering analyses of these populations were displayed in Figure 4, and the population names were marked at the top of the figure. When populations are far apart in geographic distances, individuals of these populations generally have different membership coefficients in deductive clustering. Clustering analyses showed that the color compositions of the Chinese Mongolian group were more similar to those of the East Asian populations than those of other intercontinental populations at *K* = 2 ‐ 7, which further indicated that the population structure of the Chinese Mongolian and East Asian populations was similar.

## 4. Discussion

In this study, we assessed genetic polymorphisms and forensic efficiencies of 35 InDels in the Chinese Mongolian group. Moreover, genetic relationships between the studied group and the other reference populations were explored based on these 35 InDels. Obtained cumulative PE and combined DP values of 35 InDel loci were 0.99925 and 0.9999999999999904 in the Chinese Mongolian group, indicating that these 35 InDel loci can be used as a valid tool for forensic individual identifications and as an assistant system for paternity testing. The results of PCA, phylogenetic tree, and structure analysis showed that the genetic differentiations between Chinese Mongolian group and the East Asian populations were smaller than those between the Chinese Mongolian group and the other reference populations.

Genghis Khan established the Mongol Empire in the 13th century, which was a successful nomadic nation. The Mongol Empire's territorial expansion promoted cultural exchanges between Asia and Europe, which had a remarkable influence on the genetic structure of the Eurasian people [28]. Xinjiang Mongolian is a subgroup of the Oirats, which is a branch of Mongolian (https://en.wikipedia.org/wiki/Mongols). Wei et al. stated that the Xinjiang Mongolian group had close genetic relationships with Uyghur, Xibe, and other Chinese populations [10], which was consistent with the historical record of Xinjiang Mongolian geographical migration. A previous study showed that the genetic structure of Mongolian was similar to that of CHB, JPT, and other East Asian populations [29]; besides, our result was also consistent with the results of Mei et al. which showed that Kazak group had a closer genetic relationship with the Mongolian group [30].

Our study validated the forensic applicability of these 35 InDel loci in the Xinjiang Mongolian group. In order to further carry out the population study and explain the origin of the Mongolian group, it is necessary to further evaluate the genetic characteristics of the Mongolian group by using other genetic markers like AIM, mitochondrial markers, and so on.

## 5. Conclusion

This study evaluated forensic efficiencies of a set of novel 35 InDels and assessed the genetic structure of the Chinese Mongolian group based on these InDels. The results of forensic value evaluation indicated that this system of 35 InDels was efficient enough to forensic human identifications in the Mongolian group. And the results of the population genetic analyses indicated that the genetic relationships between the Chinese Mongolian and East Asian populations were relatively close, followed by Kazak group. In a word, these results enrich the Mongolian group data and lay the basis of forensic applications of these 35 InDels in the Mongolian group.

## Figures and Tables

**Table 1:** Detailed information of 27 reference populations used in this study.

Populations	Abbreviations	Continents	Sample sizes	Sources
African Caribbean in Barbados	ACB	Africa	96	1000 genomes project phase 3
African Ancestry in Southwest USA	ASW	Africa	61	1000 genomes project phase 3
Esan in Nigeria	ESN	Africa	99	1000 genomes project phase 3
Gambian in Western Division, Mandinka	GWD	Africa	113	1000 genomes project phase 3
Luhya in Webuye, Kenya	LWK	Africa	99	1000 genomes project phase 3
Mende in Sierra Leone	MSL	Africa	85	1000 genomes project phase 3
Yoruba in Ibadan, Nigeria	YRI	Africa	108	1000 genomes project phase 3
Colombian in Medellin, Colombia	CLM	America	94	1000 genomes project phase 3
Mexican ancestry in Los Angeles, California	MXL	America	64	1000 genomes project phase 3
Peruvian in Lima, Peru	PEL	America	85	1000 genomes project phase 3
Puerto Rican in Puerto Rico	PUR	America	104	1000 genomes project phase 3
Chinese Dai in Xishuangbanna, China	CDX	East Asia	93	1000 genomes project phase 3
Han Chinese in Beijing, China	CHB	East Asia	103	1000 genomes project phase 3
Southern Han Chinese, China	CHS	East Asia	105	1000 genomes project phase 3
Japanese in Tokyo, Japan	JPT	East Asia	104	1000 genomes project phase 3
Kinh in Ho Chi Minh City, Vietnam	KHV	East Asia	99	1000 genomes project phase 3
Utah residents with Northern and Western European ancestry	CEU	Europe	99	1000 genomes project phase 3
Finnish in Finland	FIN	Europe	99	1000 genomes project phase 3
British in England and Scotland	GBR	Europe	91	1000 genomes project phase 3
Iberian populations in Spain	IBS	Europe	107	1000 genomes project phase 3
Toscani in Italy	TSI	Europe	107	1000 genomes project phase 3
Gujarati Indian in Houston, TX, USA	GIH	South Asia	103	1000 genomes project phase 3
Indian Telugu in the UK	ITU	South Asia	102	1000 genomes project phase 3
Punjabi in Lahore, Pakistan	PJL	South Asia	96	1000 genomes project phase 3
Sri Lankan Tamil in the UK	STU	South Asia	102	1000 genomes project phase 3
Bengali in Bangladesh	BEB	South Asia	86	1000 genomes project phase 3
Chinese Kazak	XJK	From China	510	Our lab

**Table 2:** Allelic frequencies and forensic parameters of the 35 InDel loci in the Chinese Mongolian group (*n*=110).

Loci	+	−	Ho	He	PIC	DP	PE	MP	TPI	*P* values
rs2308194	0.5182	0.4818	0.5636	0.5016	0.3747	0.5864	0.2495	0.4136	1.1458	0.2270
rs3067194	0.5545	0.4455	0.4727	0.4963	0.3720	0.6316	0.1647	0.3684	0.9483	0.6740
rs3028455	0.5136	0.4864	0.6273	0.5019	0.3748	0.5367	0.3249	0.4633	1.3415	0.0130
rs10629077	0.7773	0.2227	0.3545	0.3478	0.2863	0.5122	0.0886	0.4878	0.7746	1.0000
rs5846092	0.5545	0.4455	0.5818	0.4963	0.3720	0.5681	0.2696	0.4319	1.1957	0.1000
rs3082950	0.6000	0.4000	0.4727	0.4822	0.3648	0.6175	0.1647	0.3825	0.9483	0.8390
rs4210	0.5455	0.4545	0.4545	0.4981	0.3729	0.6405	0.1507	0.3595	0.9167	0.4570
rs4024564	0.4955	0.5045	0.4455	0.5022	0.3750	0.6478	0.1441	0.3522	0.9016	0.2620
rs3040095	0.4545	0.5455	0.5455	0.4981	0.3729	0.5950	0.2305	0.4050	1.1000	0.3620
rs1160964	0.4955	0.5045	0.4818	0.5022	0.3750	0.6336	0.1721	0.3664	0.9649	0.6990
rs1610945	0.4909	0.5091	0.4545	0.5021	0.3749	0.6445	0.1507	0.3555	0.9167	0.3460
rs16678	0.5091	0.4909	0.4727	0.5021	0.3749	0.6374	0.1647	0.3626	0.9483	0.5630
rs25570	0.4409	0.5591	0.5545	0.4953	0.3715	0.5863	0.2398	0.4137	1.1224	0.2520
rs16646	0.4864	0.5136	0.5909	0.5019	0.3748	0.5668	0.2801	0.4332	1.2222	0.1050
rs3066543	0.3864	0.6136	0.4636	0.4763	0.3618	0.6154	0.1576	0.3846	0.9322	0.8320
rs3029940	0.4818	0.5182	0.4909	0.5016	0.3747	0.6288	0.1797	0.3712	0.9821	0.8450
rs3839237	0.5545	0.4455	0.5455	0.4963	0.3720	0.5932	0.2305	0.4068	1.1000	0.3420
rs5882232	0.3773	0.6227	0.5545	0.4720	0.3595	0.5631	0.2398	0.4369	1.1224	0.0680
rs3057689	0.5591	0.4409	0.5182	0.4953	0.3715	0.6084	0.2039	0.3916	1.0377	0.6850
rs66502133	0.4318	0.5682	0.4455	0.4929	0.3703	0.6385	0.1441	0.3615	0.9016	0.3480
rs3831219	0.4273	0.5727	0.5091	0.4917	0.3697	0.6098	0.1956	0.3902	1.0185	0.8590
rs34224758	0.4136	0.5864	0.5364	0.4873	0.3674	0.5899	0.2213	0.4101	1.0784	0.3300
rs5803454	0.4545	0.5455	0.4909	0.4981	0.3729	0.6253	0.1797	0.3747	0.9821	1.0000
rs3054057	0.9318	0.0682	0.1182	0.1276	0.1190	0.2243	0.0114	0.7757	0.5670	0.4010
rs61681053	0.5045	0.4955	0.5364	0.5022	0.3750	0.6048	0.2213	0.3952	1.0784	0.5580
rs10556291	0.5000	0.5000	0.4727	0.5023	0.3750	0.6375	0.1647	0.3625	0.9483	0.5770
rs10637537	0.5318	0.4682	0.5182	0.5002	0.3740	0.6134	0.2039	0.3866	1.0377	0.8460
rs10609615	0.4864	0.5136	0.4455	0.5019	0.3748	0.6474	0.1441	0.3526	0.9016	0.2640
rs2307433	0.4227	0.5773	0.5182	0.4903	0.3690	0.6035	0.2039	0.3965	1.0377	0.5820
rs141749783	0.4045	0.5955	0.5000	0.4840	0.3657	0.6068	0.1875	0.3932	1.0000	0.8490
rs139995318	0.4591	0.5409	0.5182	0.4989	0.3733	0.6121	0.2039	0.3879	1.0377	0.6920
rs4189	0.5727	0.4273	0.4545	0.4917	0.3697	0.6340	0.1507	0.3660	0.9167	0.4240
rs6480	0.6500	0.3500	0.3727	0.4571	0.3515	0.6193	0.0982	0.3807	0.7971	0.0620
rs5840847	0.5545	0.4455	0.4909	0.4963	0.3720	0.6235	0.1797	0.3765	0.9821	1.0000
rs371194629	0.3682	0.6318	0.4091	0.4674	0.3570	0.6233	0.1195	0.3767	0.8462	0.2070

*Note.* +, frequency of insertion allele; −, frequency of deletion allele; Ho, observed heterozygosity; He, expected heterozygosity; PIC, polymorphism information content; DP, discrimination power; PE, probability of exclusion; MP, match probability; TPI, typical paternity index; *P* values, *P* values for Hardy–Weinberg equilibrium tests.

## Data Availability

The data used to support the findings of this study are available from the corresponding author upon request.

## Supplementary Materials

Supplementary Materials Supplementary Figure 1: the geographic localization of the Chinese Mongolian and 27 reference groups. Supplementary Figure 2: principle component analysis of the Chinese Mongolian group and the 27 reference populations at individual levels based on PLINK software (version 1.90) and ggplot 2 package (version 3.2.0) of *R* software (version 3.4.5). The African individuals are represented by blue dots, the American individuals are represented by deep green dots, the East Asian individuals are represented by light green dots, the European individuals are represented by red dots, the Kazak individuals are represented by grey dots, the South Asian individuals are represented by purple dots, and the studied Mongolian individuals are represented by yellow dots. Supplementary Figure 3: the estimated probability value (a) and Delta *K* (b) at each *K* value. Supplementary Table 1: the pairwise *P* values of linkage disequilibrium (LD) tests among these 35 InDel loci in the Chinese Mongolian group. Supplementary Table 2: absolute values of insertion allelic frequency differences (δ) between the studied Mongolian group and the other reference intercontinental populations. Supplementary Table 3: the pairwise *D_A_* values of the Chinese Mongolian group and the 27 reference populations based on a panel of 35 InDels. Supplementary Table 4: the pairwise *Fst* values of the Chinese Mongolian group and the 27 reference populations based on a panel of 35 InDels.
