# Supplementary figures and images for: Genetic Polymorphisms and Forensic Efficiencies of a Set of Novel Autosomal InDel Markers in a Chinese Mongolian Group
Source: Biomed Res Int. 2020 Jan 7;2020:3925189. doi: 10.1155/2020/3925189 (PMC6970480; doi:10.1155/2020/3925189)

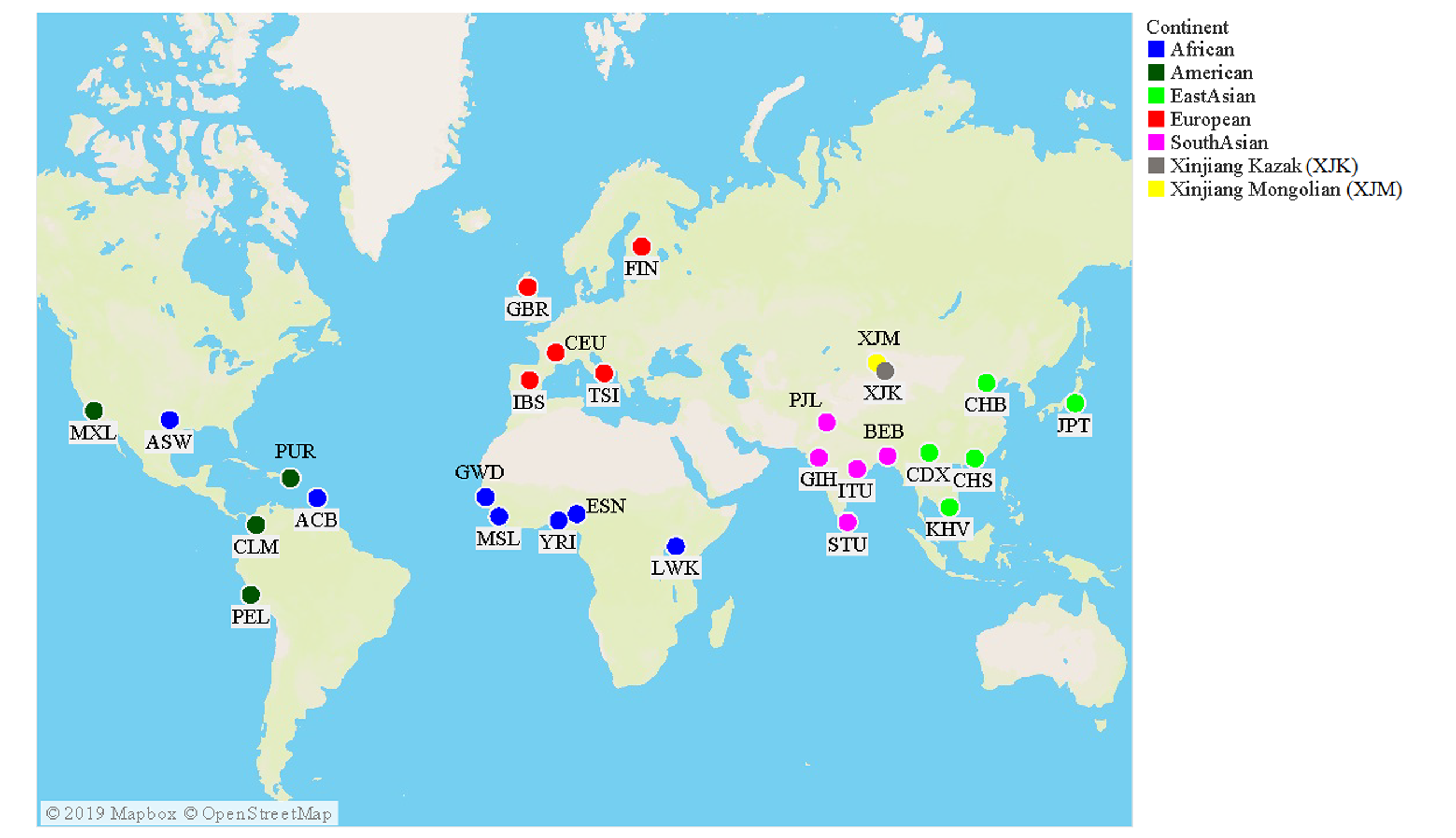

Supplement: Supplementary Materials — Supplementary Figure 1: the geographic localization of the Chinese Mongolian and 27 reference groups. Supplementary Figure 2: principle component analysis of the Chinese Mongolian group and the 27 reference populations at individual levels based on PLINK software (version 1.90) and ggplot 2 package (version 3.2.0) of R software (version 3.4.5). The African individuals are represented by blue dots, the American individuals are represented by deep green dots, the East Asian individuals are represented by light green dots, the European individuals are represented by red dots, the Kazak individuals are represented by grey dots, the South Asian individuals are represented by purple dots, and the studied Mongolian individuals are represented by yellow dots. Supplementary Figure 3: the estimated probability value (a) and Delta K (b) at each K value. Supplementary Table 1: the pairwise P values of linkage disequilibrium (LD) tests among these 35 InDel loci in the Chinese Mongolian group. Supplementary Table 2: absolute values of insertion allelic frequency differences (δ) between the studied Mongolian group and the other reference intercontinental populations. Supplementary Table 3: the pairwise DA values of the Chinese Mongolian group and the 27 reference populations based on a panel of 35 InDels. Supplementary Table 4: the pairwise Fst values of the Chinese Mongolian group and the 27 reference populations based on a panel of 35 InDels. [file 3925189.f1.zip › 3925189.f1/Supplementary Figure 1.tif]

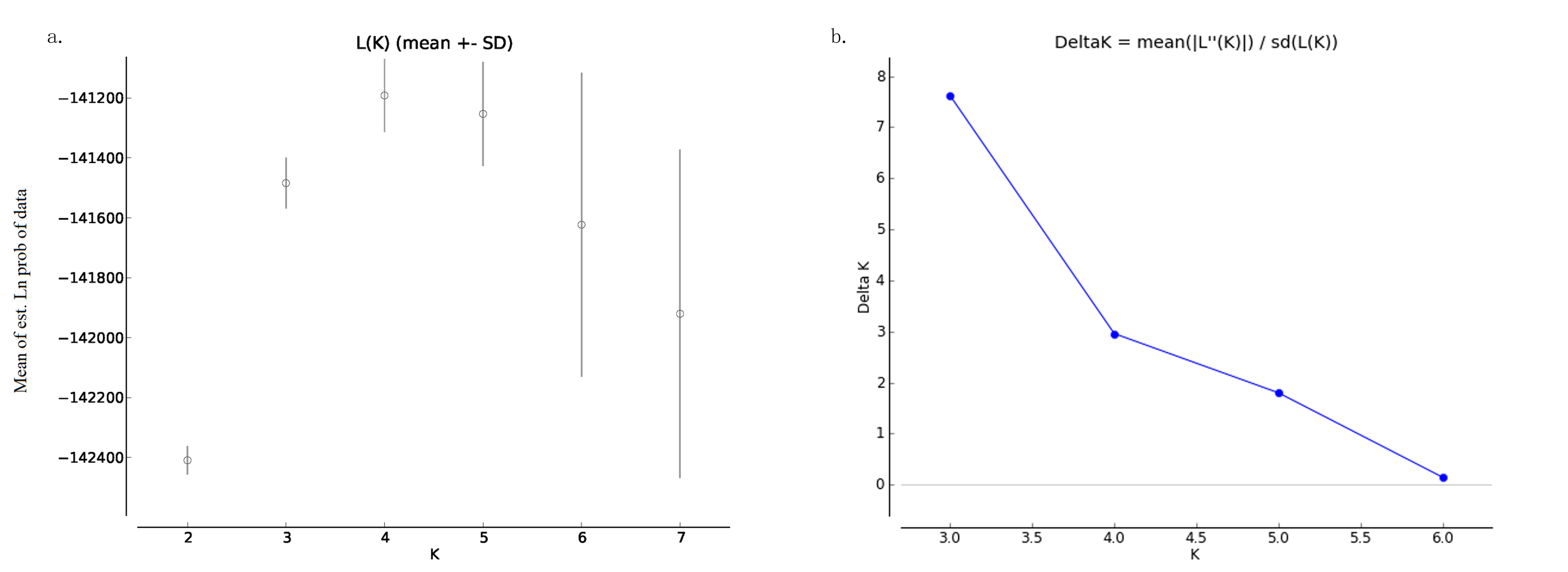

Supplement: Supplementary Materials — Supplementary Figure 1: the geographic localization of the Chinese Mongolian and 27 reference groups. Supplementary Figure 2: principle component analysis of the Chinese Mongolian group and the 27 reference populations at individual levels based on PLINK software (version 1.90) and ggplot 2 package (version 3.2.0) of R software (version 3.4.5). The African individuals are represented by blue dots, the American individuals are represented by deep green dots, the East Asian individuals are represented by light green dots, the European individuals are represented by red dots, the Kazak individuals are represented by grey dots, the South Asian individuals are represented by purple dots, and the studied Mongolian individuals are represented by yellow dots. Supplementary Figure 3: the estimated probability value (a) and Delta K (b) at each K value. Supplementary Table 1: the pairwise P values of linkage disequilibrium (LD) tests among these 35 InDel loci in the Chinese Mongolian group. Supplementary Table 2: absolute values of insertion allelic frequency differences (δ) between the studied Mongolian group and the other reference intercontinental populations. Supplementary Table 3: the pairwise DA values of the Chinese Mongolian group and the 27 reference populations based on a panel of 35 InDels. Supplementary Table 4: the pairwise Fst values of the Chinese Mongolian group and the 27 reference populations based on a panel of 35 InDels. [file 3925189.f1.zip › 3925189.f1/Supplementary Figure 3.tif]
